# The Status of Medical Student Education in Pregnancy Options Counseling: a Review

**DOI:** 10.1007/s40670-021-01368-x

**Published:** 2021-09-08

**Authors:** Lauren E. Farmer, Camille A. Clare

**Affiliations:** 1 Department of Obstetrics & Gynecology, Duke University School of Medicine, 201 Trent Dr. 203 Baker House, Durham, NC 27710 USA; 2 Department of Obstetrics and Gynecology, SUNY-Downstate Health Sciences University, Brooklyn, NY USA

**Keywords:** Pregnancy options counseling, Medical student education, OSCE, Student competency, Medical education

## Abstract

**Background:**

The Association of Professors of Gynecology and Obstetrics (APGO) has acknowledged the importance of pregnancy options counseling by listing it as a “shows how” skill for all undergraduate medical students. Unfortunately, there is no standard curriculum utilized to teach medical students pregnancy options counseling or to assess skill sustainability over time.

**Objectives:**

To review and summarize the literature on pregnancy options counseling in undergraduate medical education.

**Methods:**

We performed a structured literature review searching Google Scholar, PubMed, and EMBASE for articles between 2000 and February 2020. Inclusion criteria were English language studies of M. D. and D.O. programs in North America with a discussion of pregnancy options counseling as it relates to medical student education.

**Results:**

There is a small but growing body of literature on pregnancy options counseling in medical student education. The common themes across the 17 papers reviewed include the status of pregnancy options counseling in undergraduate medical education, barriers to teaching options counseling, the timing of education, utilization of the options counseling Objective Structured Clinical Examination (OSCE), learner challenges, and novel strategies for implementing education in options counseling and subsequent learning outcomes.

**Conclusions:**

There is no standardized pregnancy options counseling curriculum in undergraduate medical education (UME). The landscape in which this important skill is being taught is one of random, insufficient, and uncoordinated curricular interventions. This is the only review on this subject, making it a unique summary on pregnancy options counseling in UME.

## Introduction

Non-directive pregnancy options counseling is an important skill for medical students to learn as physicians of multiple specialties will relay the news of a positive pregnancy test; competency in this conversation improves patient health. Options counseling is a discussion between a provider and a patient about all possible options that a patient may choose for their pregnancy. The standard discussion includes non-coercive, non-biased counseling on both information about and access to resources for continuing a pregnancy and parenting, abortion, and adoption. Comfort with this discussion is important as unplanned pregnancy is common and patients may seek advice from their providers. In 2011, nearly half (45% or 2.8 million) of the 6.1 million pregnancies in the USA each year were unintended [1]. Options counseling at the time of positive pregnancy test is considered a patient-centered approach that is best practice for supporting pregnant persons in their pregnancy decisions [2].

Options counseling has positive implications for women’s health including reducing delays in decision-making and the potential to reduce a patient’s risk for later-term abortion procedures, in addition to reducing delays in prenatal care [3]. The goal of options counseling is to maximize the likelihood that a patient will leave an encounter following a positive pregnancy test empowered to seek safe and effective care [4]. In addition, medical providers trained in options counseling are in unique positions to identify reproductive coercion (a collection of behaviors intended to pressure or coerce a partner into initiating, keeping, or terminating a pregnancy) and intimate partner violence (IPV), which are associated with unintended pregnancy [5]. Comprehensive non-directive options counseling at the time of an unplanned positive pregnancy test is an appropriate time for education and harm reduction [2].

The Turnaway Study, a prospective longitudinal study from 2008 to 2010 that aimed to describe the mental health, physical health, and socioeconomic consequences of receiving abortion compared to carrying an unwanted pregnancy to term, found serious consequences when women were denied a wanted abortion [6]. Pregnancy options counseling is the starter conversation that connects the patient with the information and resources to pursue the option of her choice. Consequences to not obtaining these resources and not receiving desired abortion care are as follows: patients are more likely to stay tethered to abusive partners and suffer anxiety, less likely to have aspirational plans for the coming year, and more likely to experience serious pregnancy complications [6].

The Association of Professors of Gynecology and Obstetrics (APGO) has acknowledged the importance of pregnancy options counseling by listing it as a “shows how” skill for all undergraduate medical students [7]. Unfortunately, there is no standard curriculum utilized to teach medical students pregnancy options counseling or to assess skill sustainability over time. The objective of this review is to summarize the literature on pregnancy options counseling in undergraduate medical education including the teaching methods and outcomes, barriers to education, and prevalence.

## Methods

A literature review was performed using the databases of PubMed, EMBASE, and Google Scholar from 2000 to 2020. The keywords of “non-directive pregnancy options counseling,” “pregnancy options counseling,” “options counseling,” “counseling,” “pregnancy,” “clinical clerkship,” “medical school,” “medical student education,” “OSCE,” and “student competency” and variations of combinations of these keywords were utilized for the search. A modified PRISMA method was used [8]. PRISMA stands for Preferred Reporting Items for Systematic Reviews and Meta-Analyses [8]. It is an evidence-based minimum set of items aimed at helping authors report a wide array of systematic reviews and meta-analyses that assess the benefits and harms of interventions [8]. PRISMA uses a checklist. Our study was designed as a PRISMA review and met most checklist items; however, we did not perform a risk of bias assessment, nor did we perform a certainty assessment as it is necessary to meet the criteria to be a PRISMA review. This was since the studies assessed did not have congruent primary nor secondary outcomes.

Data extraction was performed independently by a primary author (L.E.F.). The data extraction variables included study authors, year of publication, description of intervention (content, timing, and participants), intervention goals, study design (quantitative and/or qualitative), main findings, assessment tool used, and opportunities and challenges identified. A secondary author (C.A.C.) confirmed the elements of the data extraction.

## Results

**Fig. 1:**
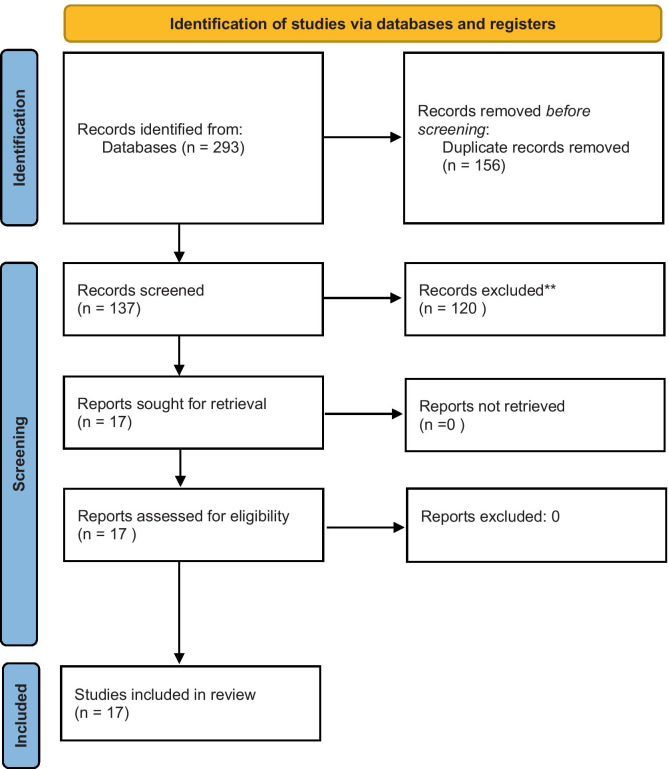
PRISMA search

A total of 293 articles were identified from the literature search (see Fig. 1 for the PRISMA search results). After the removal of duplicates, 137 articles remained for review. The inclusion criteria were English language; population of interest of medical students in M.D. and D.O. programs in the USA and Canada; a discussion of pregnancy options counseling, specifically within undergraduate medical education in the body of the paper; and peer-reviewed or expert opinion papers. If papers did not include the above criteria, they were excluded. All included papers’ citation lists were searched for titles that may have been missed in the initial search, and all eligible papers were correctly identified. Only 17 articles met the criteria.

In a study done on US and Canadian medical schools, Steinauer et al. found that pregnancy options counseling did not appear in over 50% of curricula [9]. For the most part, the settings in which elective abortion and contraception are taught to medical students are elective courses for a few students [9]. Minimal discussions are integrated into a few required preclinical courses such as physiology and pharmacology [9]. There is no standardized method for testing student competency [9]. Consequently, non-directive pregnancy options counseling is absent from many medical students’ education and very possibly from their future practice, which has implications for patient care [4, 9]. In a survey of 126 accredited US medical schools, Espey et al. found that in order to meet the APGO objectives for competence, a comprehensive curricular emphasis on all three options (parenting, abortion, and adoption) is required [10].

Eleven of 17 papers discussed various educational interventions to supplement existing medical student education in pregnancy options counseling.

### Instructional Methods

In a knowledge assessment survey of medical students, Cessford et al. found that students are often misinformed about topics in family planning, which may impact future care [11]. For example, more than 40% of surveyed students were misinformed about parental permission laws and assumed that they were required to tell a parent their child was planning to terminate a pregnancy [11]. Misinformation violates a patient’s right to make informed medical decisions including about their pregnancy options and is the consequence of inadequate education, demonstrating a need for increased attention to these subjects [11].

In an effort to offer medical student education in family planning, some schools have implemented the use of electives. Research has shown that 26% of US medical schools offer a family planning elective, which is used to fill in gaps in their curriculum [12]. In a 2015 study by Veazey et al. the authors explored students’ motivations for pursuing family planning electives throughout the USA [12]. They performed a qualitative study with semi-structured interviews of 29 students, representing 14 institutions across the USA. They found that proficiency in options counseling was one of the top competencies desired and gained by students taking family planning electives [12].

Lupi et al. (2009) described a faculty-run workshop, utilizing “helping trios” in which students took turns with different roles including patient, physician, and evaluator [13]. A student was assigned the role of patient to answer questions about how to proceed following an unplanned pregnancy, while another student assumed the role of physician, and was able to practice facilitating the conversation on the delivery of the news of an unplanned pregnancy. They found that among their cohort of 140 students, only 37% of students found that helping trios positively improved their comfort with options counseling [13]. However, the exercise was considered emotionally valuable by 95% of students [13]. It has been previously established that role play in education may have the ability to improve the student understanding of the patient experience [14]. This understanding may provide the basis for increased empathy and develop a students’ ability to communicate in a patient-centered manner [14].

In a study by Shaddeau et al. 135 students participated in a 1 h small group session, or “Novel Patient Interaction” (NPI) with a patient who discussed her own experience with her decision to have an abortion [15]. Students participated in a standardized patient exam that utilized the pregnancy options counseling, Objective Structured Clinical Exam (OSCE), before and after their experience. Ultimately, the results showed that this 1 h patient experience did not improve students’ performance on the OSCE; however, students were described as seeming more comfortable discussing pregnancy options compared to the control group [15].

In a recent study published in 2019 by Rivlin et al. students at Columbia University Medical Center were randomized either to a 2 h narrative medicine workshop or to a control intervention. Narrative medicine (NM) is the practice of engaging with illness through stories of treating patients as more than the sum of their symptoms, but rather as humans with complex histories [16]. NM has been used in medical education to promote self-reflection [16]. Students participated in reading and reflective writing exercises addressing varying perspectives on pregnancy and were assessed via the pregnancy counseling OSCE. The authors concluded that narrative medicine can be an effective tool for teaching pregnancy options counseling in medical education [16].

In 2019, Pomerantz et al. reported introducing a problem-based learning module (PBL) to second- and third-year medical students at the University of Louisville School of Medicine. Learners were presented with a case and then asked to develop objectives to research for which they convened with facilitators 1 week later to discuss [17]. The authors noted that only 50% of faculty facilitators reported feeling adequately prepared to facilitate discussions surrounding pregnancy termination [17]. They concluded that a PBL provided students with tools to better counsel patients seeking abortion and that the PBL increased students’ knowledge [17]. However, they would improve their educational session by implementing a formal didactic in the future [17]. Facilitator training was a possible barrier to this exercise [17].

Students from the Warren Alpert Medical School of Brown University in Providence, RI, organized an elective at their institution, which was run as a weekly lecture series and included a half-day experience at a local abortion clinic [18]. They assessed students via verbal feedback and an anonymous survey. In general, the more interactive sessions and clinical experiences were more highly rated [18]. Their experience highlighted barriers for using student-run electives, citing challenges for the sustainability of student electives and the logistical barriers including overcoming unsupportive administrations [18].

The largely medical student-run nonprofit organization, Medical Students For Choice (MSFC), provides externships for select students interested in advancing their skills in family planning, including a month-long clinical experience [19]. During the clinical experience, students are exposed to first trimester abortions and some second trimester abortions [20]. Eighty-three percent of students surveyed gained exposure or training in pregnancy options counseling with a proportion of students feeling knowledgeable enough to discuss abortion with patients; that doubled from 46.5 to 93.2% [3]. This program could be a model for preceptorships for students during their undergraduate medical education and was specifically successful in increasing students’ knowledge of abortion and comfort with counseling [3]. There are no other examples in the literature of institutions that provide externships for students to learn pregnancy options counseling.

### Assessment Methods and Outcomes

An important advancement to the education of medical students in options counseling was an assessment created by Lupi et al. that is, an OSCE for pregnancy options counseling [20]. This assessment was utilized in five of the quantitative studies reviewed. It has been made available for public education use on MedEd PORTAL (https://www.mededportal.org/). Since it was originally published, the OSCE assessment tool has been validated and the content has been enhanced through input from five family medicine physicians [20, 21]. It has been regularly downloaded from MedEd PORTAL since its publication in 2012 and has been found to be an acceptable and reliable way to evaluate student training in options counseling. It also serves as a tool for feedback and continuing education on the subject [22]. It may be utilized by institutions with limited resources due to its accessibility [22].

Prior clinical experience significantly improved student performance in options counseling [23, 24]. Sixty-two percent of those who reported a prior clinical experience observing or participating in options counseling achieved student-level competency, while only 38% of their peers without clinical experience achieved competency (*p* = 0.09) [23]. In an additional study, utilizing an online module and simulation, students with prior clinical experience significantly outperformed those without on the global rating scale with mean scores of 3.1 compared to 2.7, respectively (*p* = 0.44) [24].

### Barriers to Inclusion of Pregnancy Options Counseling in Undergraduate Medical Education

There are multiple barriers to the inclusion of pregnancy options counseling in medical education. These include time constraints on medical curriculum, conscientious refusal in medicine, lack of administrative support, and the challenge of sustaining electives, especially when organized by students [13]. In the USA, a majority of states have enacted “conscious clauses,” protecting providers and/or institutions that refuse to participate in objectionable interventions [13]. These have been almost entirely in response to the public focus on abortion [25]. Students who decline to participate in abortion-related care either will not observe or participate in clinical encounters in which the skills necessary for options counseling will be modeled or practiced [7]. An integrated family planning curriculum with the use of a variety of methods like lectures, tutorial cases, and essay questions would not allow students the same opportunity to opt-out and may be a solution to broadening student competency in family planning and options counseling [26].

The conversation of adoption as an option for pregnancy was cited in multiple papers as being a challenge for students [15, 20, 27]. For example, in a study by Shaddeau et al. in 2015 on novel patient interactions, more than half (54%) of the intervention group and 67% of the controls had “unsatisfactory encounters,” almost exclusively due to the omission of adoption [15]. The difference in students’ ability to explore feelings about adoption, compared with pregnancy continuation, suggests the possibility of personal bias and discomfort that may undermine professional care [27]. In addition, Lupi et al. noted that to improve options counseling, only 48% of students addressed adoption before workshop participation with a non-significant improvement to 63% after workshop participation (*p* = 0.16) [23]. Without a formal integration of adoption into undergraduate medical education curriculum, medical students may continue to overlook adoption as an option when caring for patients with unintended pregnancies [15].

In a study by Suchman et al. students routinely left discussions of intimate partner violence (IPV) and reproductive coercion out of their options counseling when assessed [14]. In addition, Lupi et al. cited that there was greater learner difficulty in asking about reproductive coercion [13, 22, 24]. Low rates of assessing IPV and reproductive coercion risk are reasons to increase education in these areas. To address the gap in these important topics, in 2016, Lupi et al. enhanced the original pregnancy options counseling OSCE by adding assessments for IPV and reproductive coercion [21].

## Discussion and Conclusion

There have been multiple efforts by various institutions, faculty, and students over the last 15 years to supplement medical student education in pregnancy options counseling. Some schools offer electives in family planning for interested students, while other educational modalities used include standardized patients, real patient experiences, narrative medicine, teaching modules, and workshops. Educational experiences have been implemented at all levels of undergraduate medical education including the preclinical years, and clinical clerkships, specifically the family medicine and obstetrics and gynecology clerkships. Additionally, interested students have taken advantage of the reproductive health externship provided by Medical Students For Choice [19].

Although there has been an increase in research on pregnancy options counseling in undergraduate medical education, the landscape in which this important skill is being taught to medical students is one of random, insufficient, and uncoordinated curricular interventions. To support the learning of this skill, there is a need for coordinated education offered to all medical students, improved teaching, and clinical learning. There is an array of instructional strategies utilizing a mix of lecture (which is not sufficient for skills learning), simulation, and experiential learning. There are challenges that students have including relatively poor performance in discussions of intimate partner violence and reproductive coercion and omission of adoption as a counseling option.

Proficiency in options counseling was one of the top competencies desired and gained by students taking family planning electives [12]. Family planning electives are a self-selecting population of students who choose that these subjects are important to them. Electives are not sufficient to educate the medical student population on how to provide patient-centered non-directive pregnancy options counseling and are challenging to sustain if local administrations are not supportive. In general, if electives are used, more interactive sessions and in-person clinical experiences were more highly rated [18]. Medical Students For Choice provides a month-long clinical experience that doubled student self-reported knowledge in abortion [19]. In lieu of a formal standardized curriculum nationwide, this elective may continue to provide interested students with meaningful experience.

Of the techniques documented that have been used to teach medical students, helping trios seemed to be insufficient in terms of improving student comfort with the skill [13]. A 1 h patient experience was also not considered to be adequate to improve student performance in options counseling [15]. A problem-based learning module at the University of Louisville proved to increase student knowledge; however, a formal didactic session was recommended for the future and facilitator training was identified as a barrier [17]. Narrative medicine, as tested by researchers at Columbia University Medical Center via a 2 h workshop, seemed to improve student OSCE scores (*p* = 0.05) when students were randomized to a 2 h workshop vs. control intervention [16]. The NM group participated in reading and reflective writing exercises addressing varying perspectives on pregnancy. This 2 h session could be adapted and utilized at other institutions. The options counseling OSCE has been validated for quality. It provides an objective measurement of student performance in the skill and has been utilized by multiple institutions to assess educational interventions. Its use should be encouraged as it is the only objective validated tool to assess this important skill. It is available on the MedEd PORTAL.

Currently, there is no consensus for an optimal combination of modalities to ensure student competence and comfort in pregnancy options counseling. Though the pregnancy options counseling OSCE has been cited as a validated assessment tool in undergraduate medical education, there has been little to no discussion on the education and experience required to achieve skill sustainability, specifically maintained competency over time. In addition, standardized curriculum necessary to prepare students for OSCE level competency has yet to be defined.

The limitations of this review include the relatively small body of literature on the subject (*n* = 17) and lack of ability to find statistically significant relationships from the studies. A strength of this review is that it is a unique summary on pregnancy options counseling in undergraduate medical education. This review may serve as a guide to accessing the emerging body of literature on pregnancy options counseling in medical student education, making these resources more accessible to those committed to expanding medical student education in options counseling.

Future steps to advance a family planning curriculum, specifically options counseling, should be directed at the following: (a) determining what combination of educational activities and clinical exposures is optimal for students to achieve competency in pregnancy options counseling, (b) building upon the OSCE to assess student competency in pregnancy options counseling and standardizing an assessment strategy for all medical students, (c) providing students with early clinical experience to practice options counseling and to gain exposure to abortion procedures in order to inform their counseling, (d) improving student understanding of the local adoption process so that students may counsel appropriately, and (e) improving student understanding of intimate partner violence and reproductive coercion and comfort counseling on these to.
